# How do we raise media bias awareness effectively? Effects of visualizations to communicate bias

**DOI:** 10.1371/journal.pone.0266204

**Published:** 2022-04-13

**Authors:** Timo Spinde, Christin Jeggle, Magdalena Haupt, Wolfgang Gaissmaier, Helge Giese

**Affiliations:** 1 Department of Computer and Information Science, University of Konstanz, Konstanz, Germany; 2 School of Electrical, Information and Media Engineering, University of Wuppertal, Wuppertal, Germany; 3 Department of Psychology, University of Konstanz, Konstanz, Germany; Universiti Pertahanan Nasional Malaysia, MALAYSIA

## Abstract

Media bias has a substantial impact on individual and collective perception of news. Effective communication that may counteract its potential negative effects still needs to be developed. In this article, we analyze how to facilitate the detection of media bias with visual and textual aids in the form of (a) a forewarning message, (b) text annotations, and (c) political classifiers. In an online experiment, we randomized 985 participants to receive a biased liberal or conservative news article in any combination of the three aids. Meanwhile, their subjective perception of media bias in this article, attitude change, and political ideology were assessed. Both the forewarning message and the annotations increased media bias awareness, whereas the political classification showed no effect. Incongruence between an articles’ political position and individual political orientation also increased media bias awareness. Visual aids did not mitigate this effect. Likewise, attitudes remained unaltered.

## Introduction

The Internet age has a significant impact on today’s news communication: It allows individuals to access news and information from an ever-increasing variety of sources, at any time, on any subject. Regardless of journalistic standards, media outlets with a wide reach have the power to affect public opinion and shape collective decision-making processes [1]. However, it is well known that the wording and selection of news in media coverage often are biased and provide limited viewpoints [2], commonly referred to as *media bias*. According to Domke and colleagues [3], media bias is a structural, often wilful defect in news coverage that potentially influences public opinion. Labeling named entities with terms that are ambiguous in the concepts they allude to (e.g. "illegal immigrants" and "illegal aliens" [4] or combining concepts beyond their initial contexts into figurative speech that carry a positive or negative association ("a wave of immigrants flooded the country") can induce bias. Still, the conceptualization of media bias is complex since biased and balanced reporting cannot be distinguished incisively [5]. Many definitions exist, and media bias, in general, has been researched from various angles, such as psychology [6], computer science [7], linguistics [8], economics [9], or political science [10]. Therefore, we believe advancement in media bias communication is relevant for multiple scientific areas.

Previous research shows the effects of media bias on individual and public perception of news events [6]. Since the media are citizens’ primary source of political information [11], associated bias may affect the political beliefs of the audience, party preferences [12] and even alter voting behavior [13]. Moreover, exposure to biased information can lead to negative societal outcomes, including group polarization, intolerance of dissent, and political segregation [14]. It can also affect collective decision-making [15]. The implications of selective exposure theory intensify the severity of biased news coverage: Researchers observed long ago that people prefer to consume information that fits their worldview and avoid information that challenges these beliefs [16]. By selecting only confirmatory information, one’s own opinion is reaffirmed, and there is no need to re-evaluate existing stances [17]. In this way, the unpleasant feeling of cognitive dissonance is avoided [18]. Isolation in one’s own *filter bubble* or *echo chamber* confirms internal biases and might lead to a general decrease in the diversity of news consumption [14]. This decrease is further exacerbated by recent technological developments like personalized overview features of, e.g., news aggregators [19]. How partisans select and perceive political news is thus an important question in political communication research [20]. Therefore, this study tries to test ways to increase the awareness of media bias (which might mitigate its negative impact) and the partisan evaluation of the media through transparent bias communication.

### Media bias communication

Media bias occurs in various forms, for example, whether or how a topic is reported (D’Alessio & Allen, 2000) and may not always be easy to identify. As a result, news consumers often engage with distorted media but are not aware of it and exhibit a lack of *media bias awareness* [21]. To address this issue, revealing the existence and nature of media can be an essential route to attain media bias awareness and promote informed and reflective news consumption [19]. For instance, visualizations may generally help to raise media bias awareness and lead to a more balanced news intake by warning people of potential biases [22], highlighting individual instances of bias [19], or facilitating the comparison of contents [2, 23].

Although knowledge of how to communicate media bias effectively is crucial, visualizations and enhanced perception of media bias have only played a minor role in existing research, and several approaches have not yet been investigated. Therefore, this paper tests how effectively different strategies promote media bias awareness and thereby may also help understand common barriers to informed media consumption. We selected three major methods in related work [19, 22] on the topic to further investigate them in one combined study: forewarning messages, text annotations, and political classifications. Theoretical foundations of bias messages and visualizations are yet scarce, and neither in visualization theory nor in bias theory, suitable strategies in the domain have been extensively tested.

#### Forewarning message

According to socio-psychological inoculation theory [24], it is possible to pre-emptively confer psychological resistance against persuasion attempts by exposing people to a message of warning character. It is similar to the process of immunizing against a virus by administering a weakened dose of the virus: A so-called inoculation message is expected to protect people from a persuasive attack by exposing them to weakened forms of the persuasion attempt. Due to the perceived threat of the forewarning inoculation message, people tend to strengthen their own position and are thus more resistant to influences of imminent persuasion attacks [25]. Therefore, one strategy to help people detect bias is to prepare them ahead of media consumption that media bias may occur, thereby "forewarning" them against biased language influences. Such warnings have been widely established in persuasion and shown to be effective in different applied contexts [26]. Furthermore, such warnings also seem to help not only to protect attitudes against influences but also to determine the quality of a piece of information [27–29] and communicate the information accordingly [30]. For biased language, this may work specifically by focusing the reader’s attention on a universal motive to evaluate the accuracy of information while relying on the individual’s capacity to detect the bias when encountered [30]; Bolsen & Druckman, 2015).

#### Annotations

**Fig 1:**
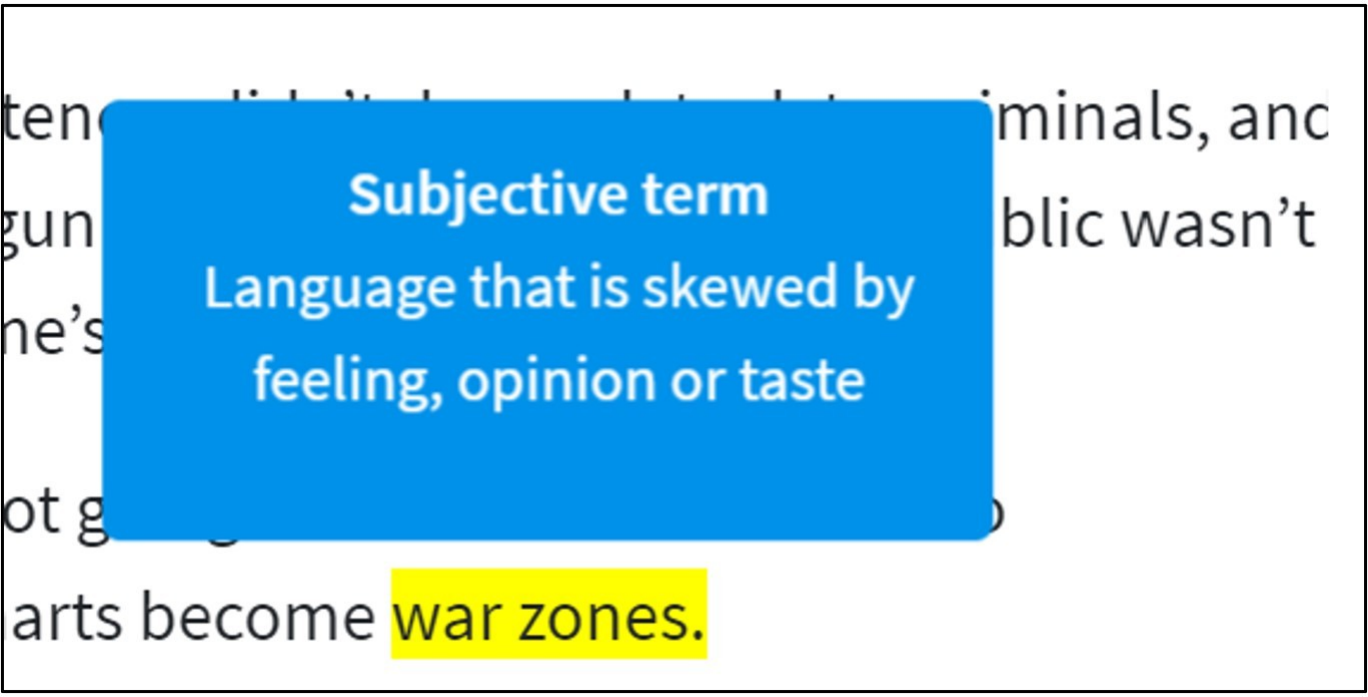
Annotation visualization. Example of the bias annotation "subjective term". Boxed annotation appeared by moving the cursor/finger over the highlighted text section.

Other than informing people in advance about bias occurrence, a further approach is to inform them during reading, thereby increasing their awareness of biased language and providing direct help to detect it in an article. Recently, there has been a lot of research on media bias from information science, but it is mainly concerned with its identification and detection [31–34]. However, whereas some research concerning the effects of visualizations of media bias in news articles to detect bias are promising (here: flagging fake news as debunked [35]) others did not find such effects, potentially also due to the technical issues in accurately annotating single articles [19]. Still, they offer a good prospect to enable higher media bias awareness and more balanced news consumption. We show our annotation visualization in Fig 1.

#### Political classification

**Fig 2:**
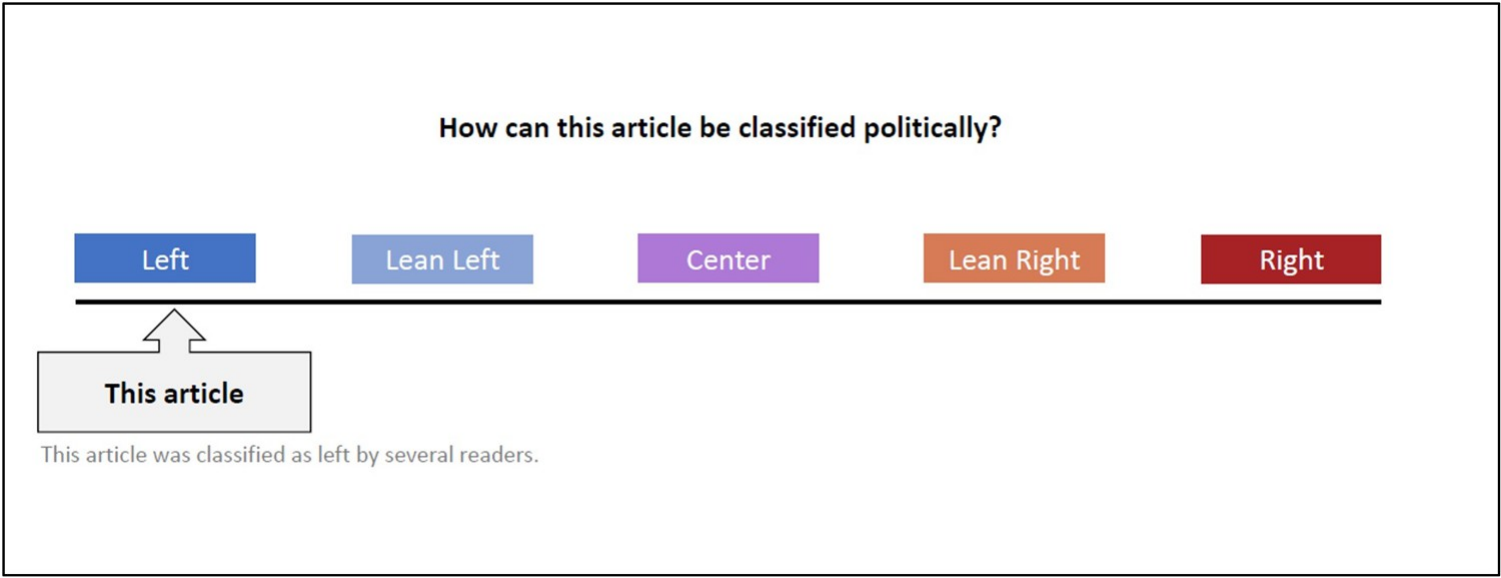
Political classification bar. Example of an article classification as being politically left-oriented.

Another attempt to raise media bias awareness is a political classification of biased material after readers have dealt with it. An and colleagues [36] proposed an ideological left-right map where media sources are politically classified. The authors suggest that showing a source’s political leaning helps readers question their attitudes and even promotes browsing for news articles with multiple viewpoints. Likewise, several other studies indicate that feedback on the political orientation of an article or a source may lead to more media bias awareness and a more balanced news consumption [19]. Additionally, exposing users to multiple diverse viewpoints on controversial topics encourages the development of more balanced viewpoints [23]. A study of Munson and colleagues (2013) further suggests that a feedback element indicating whether the user’s browsing history consists of biased news consumption modestly leads to a more balanced news consumption. Based on these findings, we will test whether the sole representation of a source’s leaning helps raise bias awareness among users on the condition that the article is classified as politically skewed. We show our political classification bar in Fig 2.

### Partisan media bias awareness

Attempts to raise media bias awareness may be further complicated by the fact that the detection of media bias and the evaluation of news seem dependent on the political ideology of the beholder [37–41]. However, this partisan effect is not only apparent in neutral reporting: It is supposed that individuals perceive biased content that corresponds to their opinion as less biased [38] and biased content that contradicts their viewpoints as more biased [41].

These findings suggest that incongruence between the reader’s position and the news article’s position may increase media bias perception of the article, whereas congruence may decrease it. Thus, partisan media consumers may engage in motivated reasoning to overcome cognitive dissonance experienced when encountering media bias in any news article generally in line with their viewpoints [42]. According to Festinger [18], cognitive dissonance is generated when a person has two cognitive elements that are inconsistent with each other. This inconsistency is assumed to produce a feeling of mental discomfort. People who experience dissonance are motivated to reduce the inconsistency because they want to avoid or reduce this negative emotion.

Furthermore, Festinger notes that exposure to messages inconsistent with one’s beliefs could create cognitive dissonance, leading people to avoid or reduce negative emotions. In line with this notion, raising media bias awareness could increase experienced cognitive dissonance and thereby lead to even more partisan ratings of bias. Another explanation of the phenomenon of partisan bias ratings is varying norms about what content is considered appropriate in media coverage dependent on one’s political identity[43]. Other researchers focus on the inattention to the quality of news and the motive to only support truthful news [44]. Both approaches lead us to expect opposite results for the partisanship of the media bias ratings with increased media bias awareness as created by our proposed visualizations: Partisanship of ratings should decrease rather than increase as people are reminded of more general norms and accuracy motives [27].

### Study aims and hypotheses

This project aims to contribute to a deeper understanding of effective media bias communication. To this end, we create a set of bias visualizations revealing bias in different ways and test their effectiveness to raise awareness in an online experiment. Following the respective literature elaborated above for each technique, we would expect enhanced media bias awareness by all visualizations:

H1a: A forewarning message prior to news articles increases media bias awareness in presented articles.
H1b: Annotations in news articles increase media bias awareness in presented articles.
H1c: A political classification of news articles increases media bias awareness in presented articles.

Another goal of this study is to understand better the reader’s political orientation in media bias awareness. In line with the findings of partisan media bias perception (hostile media effect; Vallone et al., 1985), we adopt the following hypothesis:

H2: Presented material will be rated less biased if consistent with individual political orientation.

Furthermore, we assume, following the attentional and normative explanation of partisanship in ratings rather than cognitive dissonance theory, the following effect:

H3: Bias visualizations will mitigate the effects of partisan bias ratings.

## Methods

### Participants

A total of 1002 participants from the US were recruited online via Prolific in August of 2020. A final sample of *N* = 985 was included in the analysis (51% female; *age*: *M* = 32.67; *SD* = 11.95*)*. The excluded participants did not fully complete the study or indicated that their data might not be trusted in a seriousness check. The target sample size was determined using power analysis, so that small effects (*f* = 0.10) could be found with a power of .80 [45]. The online study was scheduled to last approximately 10 minutes, for which the participants received £1.10 as payment.

### Design and procedure

The experiment was conducted online in Qualtrics (https://www.qualtrics.com). It operated with fully informed consent, adheres to the Declaration of Helsinki, and was conducted in compliance with relevant laws and institutional guidelines, including the ones of the University of Konstanz ethics board. All participants confirmed their consent in written form and were informed in detail about the study, the aim, data processing, anonymization, and other background information.

After collecting informed consent and demographic information, we conducted an initial attitude assessment which asked for their general perception of the presented topic on three dimensions and personal relevance. Next, participants read one randomly selected biased news article (either liberal or conservative), randomly supplemented by any combination of the visual aids (forewarning message, annotations, political classification). Thus, the study had a 2x2x2 *forewarning message* (yes/no) x *annotations* (yes/no) *x political map* (yes/no) between design. The article also varied between participants in both *article position* (liberal/conservative) and *article topic* (gun law/abortion) to determine the results’ partialness and generalizability. Finally, attitudes towards the topic were reassessed, followed by a seriousness check.

### Study material

#### Visual aids

*Forewarning message*. The forewarning message consisted of a short warning and was displayed directly before the news article. It reads: "***Beware of biased news coverage*. *Read consciously*. *Don’t be fooled*.** *The term ’media bias’ refers to*, *in part*, *non-neutral tonality and word choice in the news*. *Media Bias can consciously and unconsciously result in a narrow and one-sided point of view*. *How a topic or issue is covered in the news can decisively impact public debates and affect our collective decision making*." Besides, an example of one-sided language was shown, and readers were encouraged to consume news consciously.

*Annotations*. Annotations were directly integrated into the news texts. Biased words or sentences were highlighted [46], and by hovering over the marked sections, a short explanation of the respective type of bias appeared. For example, if moving the cursor over a very one-sided term, the following annotation would be displayed: "*Subjective term*: *Language that is skewed by feeling*, *opinion or taste*." Annotations were based on ratings of six members of our research group, where phrases had to be nominated by at least three raters. The final annotations can be found in the supplementary preregistration repository accompanying this article at https://osf.io/e95dh/‌?view_only=d2fb5dc‌2d64741e393b30b9ee6cc7dc1 (Non-anonymous Link is made accessible in case of acceptance). We followed the guidelines applied in existing research to teach annotators about bias and reach higher-quality annotations [47]. In future work, we will further increase the number of raters, as we address in the discussion.

*Political classification*. A political classification in the form of a spectrum from left to right indicated the source’s political ideology. It was displayed immediately after the presented article and based on the rating of the webpage Allsides.

#### Articles

We used four biased news articles that varied in topic and political position. Each participant was assigned to one article. The two topics covered were gun law and the debate on abortion, with either a liberal or conservative article position. Topics were selected because we considered them controversial issues in the United States that most people are presumably familiar with. To ensure that articles were biased, they were taken from sources deemed extreme according to the Allsides classification. Conservative texts were taken from Breitbart.com; liberal articles were from Huffpost.com and Washingtonpost.com. We also conducted a manipulation check to determine whether participants perceived political article positions in line with our assumptions: Just after reading the article, participants were asked to classify its political stance on a visual analogue scale (-5 = *very liberal* to 5 = *very conservative*). To ensure comparability, articles were shortened to approximately the same length, and respective sources were not indicated. All article texts used are listed together with their annotations in the supplementary preregistration repository accompanying this article (we show the link on the previous page).

### Measures

#### Media bias awareness

Five semantic differentials assessed media bias awareness on fairness, partialness, acceptableness, trustworthiness, and persuasiveness [48–50] on visual analogue scales ("*I think the presented news article was…*"). Media bias awareness was established by averaging the five items and recoded to range from -5 = low bias awareness to 5 = high bias awareness (*α* = .88).

#### Political orientation

The variable political orientation was measured on a visual analogue scale ranging from –5 = *very conservative* to 5 = *very liberal*), introduced with the question "*Do you consider yourself to be liberal*, *conservative*, *or somewhere in between*?" adopted by Spinde and colleagues [19, 51]. Likewise, we assessed the perceived stance of the read article on the same scale introduced with the question "*I think the presented news article was…*".

#### Attitudes towards article topic

Attitudes were assessed before and after the article presentation by a three-item semantic differential scale (*wrong*-*right*, *unacceptable*-*acceptable*, *bad*-*good*) evaluating the two topics ("*Generally*, *laws restricting abortion/ the use of guns are*. . ."; α = .99). The three items were averaged per topic to yield a score from (–5 = very conservative attitude to 5 = very liberal attitude). Besides, we assessed topic involvement by one item before the article presentation ("*To me personally*, *laws restricting the use of guns/ abortions are… irrelevant-relevant")* on a scale from –5 to 5.

### Statistical analysis

To test effects of the visual aids on media bias perception, we used ANOVAs with effect coded factors in a *forewarning message* (yes/no) x *annotations* (yes/no) *x political map* (yes/no) x2 *article position* (liberal/conservative) x2 *article topic* (gun law/abortion) between design. For analyses testing political ideology effects, this was generalized to a GLM with standardized political orientation as an additional interacting variable followed by a simple effects analysis. The same model was applied to the second attitude rating, with first attitude rating and topic involvement as covariates for attitude change. This project and the analyses were preregistered with the DOI https://osf.io/e95dh/?view_only=d2fb5dc2d64741e39‌3b30b9ee6cc7dc1 (Non-anonymous Link is made accessible in case of acceptance). All study materials, code, and data are available there.

## Results

### Manipulation check and other effects on perceived political stance of the article

**Fig 3:**
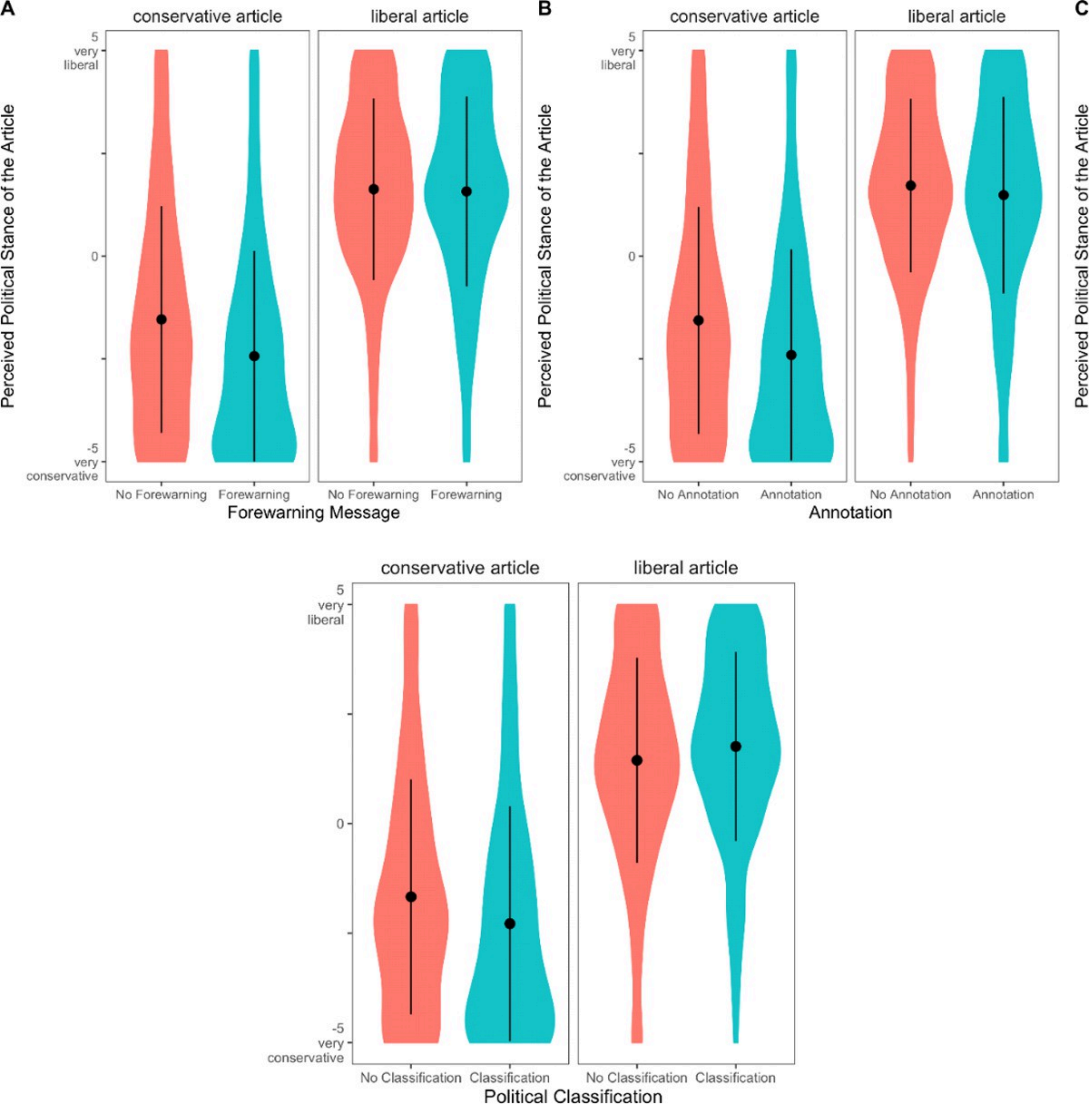
Perceived stance of conservative and liberal articles by intervention conditions. Across all conditions, liberal articles were perceived to be more liberal and conservative articles more conservative. The interventions increased the differences between the two ratings. Dots represent means, and lines are standard deviations.

Overall, the positions of the political articles were perceived as designed (*article position*: *F*(1, 953) = 528.67, *p* < .001, η_p_^2^ = .357): Articles assigned a liberal position were perceived more liberal (*M* = 1.60, *SD* = 2.70), whereas conservative articles were rated more conservative (*M* = –1.98, *SD* = 2.26). This difference between the conservative and the liberal article was more pronounced, when a forewarning message (*F*(1, 953) = 7.33, *p* = .007, η_p_^2^ = .008), annotations (*F*(1, 953) = 3.96, *p* = .047, η_p_^2^ = .004), or the political classifications were present (*F*(1, 953) = 9.12, *p* = .003, η_p_^2^ = .009; see Fig 3). The combination of forewarning and classification further increased the difference (*F*(1, 953) = 5.28, *p* = .022, η_p_^2^ = .006).

### Effects of visual aids on media bias perceptions

**Fig 4:**
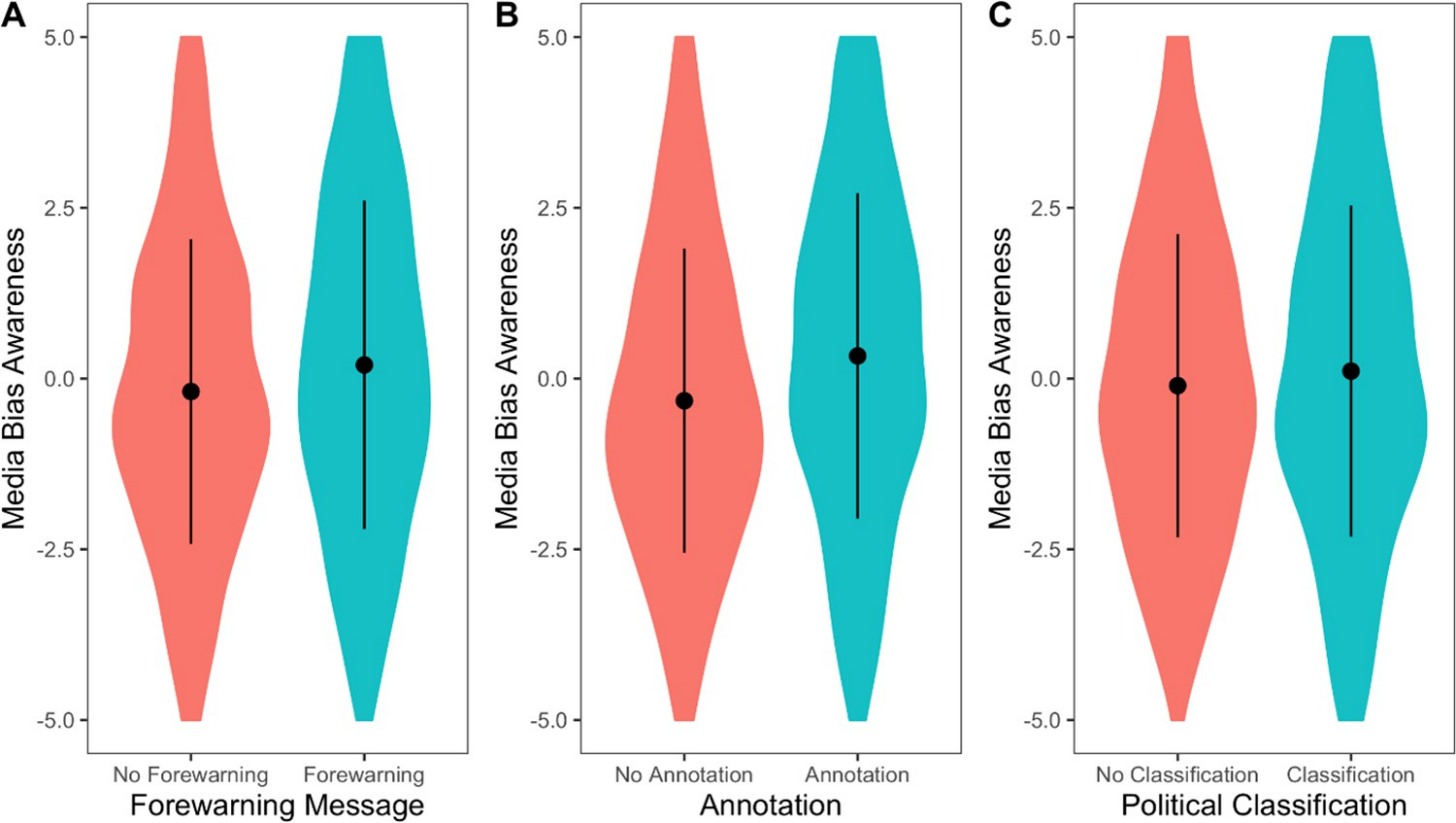
Effects of the interventions on media bias awareness. The forewarning message, as well as annotations, increased media bias awareness. Dots represent means, and lines are standard deviations.

Testing the effects of the visual aids on media bias perceptions in general, we found that both the forewarning message (*F*(1, 953) = 8.29, *p* = .004, η_p_^2^ = .009) and the annotations (*F*(1, 953) = 24.00, *p* < .001, η_p_^2^ = .025) increased perceived bias, which we show in Fig 4. However, we found no effect of the political classification (*F*(1, 953) = 2.56, *p* = .110, η_p_^2^ = .003) and no systematic higher-order interaction involving any of the manipulations (*p* ≥ .085, η_p_^2^ ≤ .003). Moreover, there were differences in media bias perceptions of the specific articles (*topic* x *article position*: *F*(1,953) = 24.44, *p* < .001, η_p_^2^ = .025). The two found main effects were by and large robust when testing it per item of the media bias perception scale (forewarning had no significant effect on partialness and persuasiveness) or in a MANOVA (*forewarning*: *F*(5, 949) = 5.22, *p* < .001, η_p_^2^ = .027; *annotation*: *F*(5, 949) = 6.25, *p* < .001, η_p_^2^ = .032).

### Partisan media bias ratings

**Fig 5:**
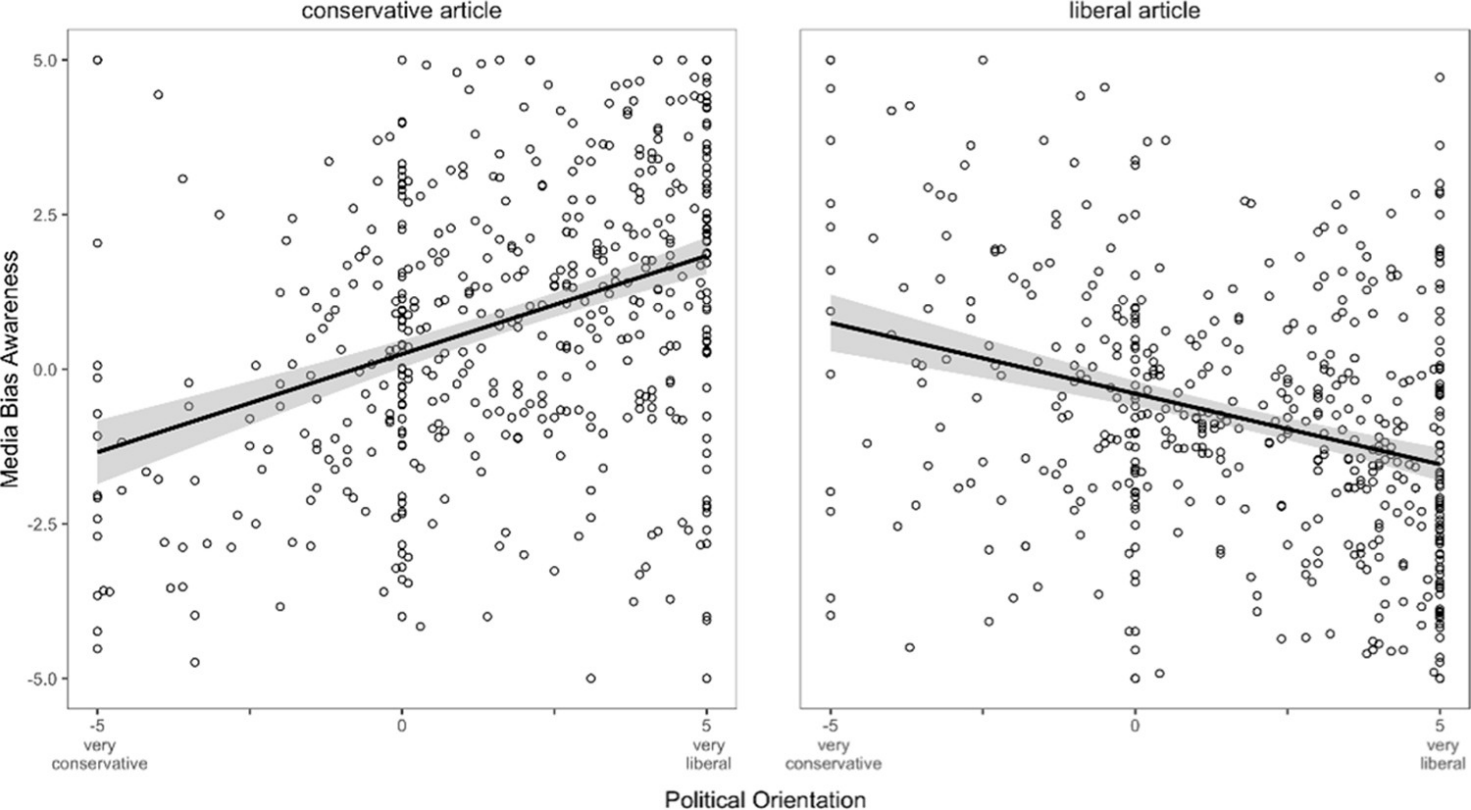
Partisan effects on media bias awareness ratings of conservative and liberal articles. Bias awareness increases when the article is not aligned with the persons’ political position. Shades show 95% confidence intervals of the regression estimation.

When considering self-indicated political orientation and its fit to the *article position*, we found that media bias was perceived less for articles consistent with the reader’s political orientation (*F*(1,921) = 113.37, *p* < .001, η_p_^2^ = .110): For conservative articles, liberal readers rated conservative articles more biased than conservative readers (β = 0.32; *p* < .001; 95%CI[0.25; 0.38]). Conversely, liberal articles were rated less biased by liberals (β = –0.20; *p* < .001; 95%CI[–0.27; –0.13]), indicating a partisan bias rating for both political isles, which we show in Fig 5.

This partisan rating of articles was unaffected by *forewarning* (*F*(1,921) = 1.52, *p* = .218, η_p_^2^ = .002), *annotations* (*F*(1,921) = 0.26, *p* = .612, η_p_^2^ < .001), and *political classification* (*F*(1,921) = 2.72, *p* = .010, η_p_^2^ = .003). Yet, with increasing liberalness of the reader, the combination of forewarning and annotation was slightly less effective on the detection of bias (*F*(1,921) = 4.19, *p* = .041, η_p_^2^ = .005). Furthermore, there were some topic-related differences irrelevant to the current hypotheses (higher bias was perceived for the gun laws articles (*topic*: *F*(1,921) = 11.32, *p* < .001, η_p_^2^ = .012) and specifically so for the liberal one (*topic* x *article position*: *F*(1,921) = 23.86, *p* < .001, η_p_^2^ = .025) with some uninterpretable minor higher order interaction (*forewarning* x *annotation* x *classification* x *political orientation* x *topic*: *F*(1,921) = 4.10, *p* = .043, η_p_^2^ = .004)).

### Effects on attitudes

By and large, attitudes on the topics were not affected by the experiment: While attitudes after reading the article were in line with prior attitudes (*F*(1,919) = 2415.42, *p* < .001, η_p_^2^ = .724) and individual political orientation (*F*(1,919) = 34.54, *p* < .001, η_p_^2^ = .036), neither *article position* (*F*(1,919) = 2.63, *p* = .105, η_p_^2^ = .003) nor any of the visual aids had any general impact (*p* ≥ .084, η_p_^2^ ≤ .003). Likewise, neither of the aids interacted with the factor *article position* (*p* ≥ .298, η_p_^2^ ≤ .001). Solely, there were some additional minor topic-specific significant effects of the annotation combined with the *forewarning* (*F*(1,919) = 4.77, *p* = .0292, η_p_^2^ = .005) and an increased liberalness of attitude with higher topic involvement (*F*(1,919) = 4.31, *p* = .038, η_p_^2^ = .005), that we want to disclose, but deem irrelevant to our hypotheses and research questions.

## Discussion

In this study, we tested different techniques to communicate media bias. Our experiment revealed that presenting a forewarning message and text annotations enhanced awareness of biased reporting, while a political classification did not. All three methods (forewarning, annotation, political classification) impacted the political ideology rating of the presented article. Furthermore, we found evidence for partisan bias ratings: Participants rated articles that agreed with their general orientation to be less biased than articles from the other side of the political spectrum. The positive effect of the forewarning message on media bias ratings, albeit small, is in line with a few other findings of successful appeals to and reminders of accuracy motives [30]. In addition, it accords with the notion that reflecting on media bias involves some efforts [44, 52], so motivating people to engage in this process can help detect bias.

Regarding the effects of in-text annotations, our finding differs from a previous study of a similar design [19], which did not identify the effect due to a lack of power and less optimal annotations. While news consumers may generally identify outright false or fake [53] news, detecting subtle biases can profit from such aids. This indicates that bias detection is far from ideal, particularly in more ambiguous cases. As in-text annotation and forewarning message effects were independent of each other, participants seemingly do not profit from the combination of aids.

On the other hand, the political classification could solely improve the detection of the political alignment of the text (which was also achieved by both other methods) but not help detecting biased language. Subsequently, the detection of biased language and media bias itself does not appear to be directly related to an article’s political affiliation.

Our study also replicates findings that the detection of media bias and fake news is affected by individual convictions [30, 40, 42]: We found that participants could detect media bias more readily if there was an incongruence between the participant’s and the article’s political ideology. Such a connection may be particularly true for detecting more subtle media biases and holding an article in high regard compared to successfully identifying outright fake news, for which a reversed effect could be found in some instances (Pennycook & Rand, 2019).

In addition, interventions were ineffective to lower such partisan effects. Similarly, attitudes remained relatively stable and were not affected by any of the visual aids. Making biased language more visible and reminding people of potential biases could apparently not help them overcome their ideology in rating the acceptance of an article when there is no clear indication that the information presented in the article is fake but solely biased. Likewise, the forewarning message successfully altered the motivation to look for biased language, but did not decrease the effects of political identity on the rating: While being able to detect the political affiliation of an article, it seems that participants were not capable of separating the stance of the article from its biased use of language, even when prompted to do so. In the same vein, effects were not more pronounced when the political classification was further visualized, potentially pointing to the notion that the stance is also detected without help (after all, while the manipulations increased the distinction between liberal and conservative articles, the article’s position was reliably identified even without any supporting material) and that partisan ratings are not a deliberate derogatory act. Furthermore, the problem of partisan bias ratings also did not increase with increased media bias awareness via the manipulations, as could have been expected by cognitive dissonance theory.

For future work, we will improve the representativeness of the surveyed sample, which limits far-reaching generalizations at this point. Additionally, we will increase the generalizability by employing articles that are politically neutral or exhibit comparatively low bias. Both forewarning and annotations may have increased ratings in this study, but it is unclear whether they also aid in identifying low-bias articles and leading to lower ratings, respectively. Improving the quality of our annotations by including more annotators is an additional step towards exhausting potential findings. We will also investigate how combinations of the visualizations and strategies work together and conduct expert interviews to determine which applications would be of interest in an applied scenario. Still, the current study shows that two of our interventions raised attention to biased language in media, giving a first insight into the yet sparsely tested field of presenting media bias to news consumers.

Furthermore, there is a great challenge in translating these experimental interventions to applications used by news consumers in the field. While forewarning messages could be implemented quite simply in the context of other media, for instance, as a disclaimer (see [30]), we hope that automated classifiers on the sentence level will prove to be an effective tool to create instant annotating aids for example as browser add-ons. Even though recent studies show promising accuracy improvements for such classifiers [31, 32], we still want to note that much research needs to be devoted to finding stable and reliable markers of biased language. Future work also has great potential to consider these strategies as teaching tools to train users in identifying bias without visual aids. This could offer a framework for a large-scale study in which additional variables measuring previous news consumption habits could be employed.

## Conclusion

In the context of our digitalized world, where news and information of differing quality are available everywhere, our results provide important insights for media bias research. In the present study, we were able to show that forewarning messages and annotations increased media bias awareness among readers in selected news articles. Also, we could replicate the well-known hostile media bias that consists of people being more aware of bias in articles from the opposing side of the political spectrum. However, our experiment revealed that the visualizations could not reduce this effect, but partisan ratings rather seemed unaffected. In sum, digital tools uncovering and visualizing media bias may help mitigate the negative effects of media bias in the future.
